# 

**Published:** 1920-03-13

**Authors:** 


### Estates of Medical Men.

Dr. C. A. Mercier, of Bournemouth, the well-known specialist in mental diseases and publicist, has left £4,829.

Mr. Robert Craig Maclagan M.D., F.R.C.P., F.R.S.E., a professor at Edinburgh University, and for over thirty years chairman of Messrs. A. B. Fleming and Co., Ltd., manufacturing chemists, of Edinburgh and London, left personal estate in the United Kingdom valued at £93,261.

### Royal Albert Edward Infirmary, Wigan.

It is proposed to place in the Wigan Infirmary a permanent memorial of all grades of the nursing staff who served in military hospitals at home or abroad during the Great War. The General Superintendent and Secretary would accordingly be very glad if those who so acted would communicate with him stating in which hospital or hospitals they served, any decorations won, and the period during which they were engaged at the Wigan Infirmary. Any information regarding others, and in particular any who may have died as a result of war service, will be also much appreciated.

### Matrons' and Sisters' Appointments.

General Hospital, Altrincham.—Miss Lornetta Roskell, A.R.R.C., has been appointed night sister. She was trained at the above institution, and has since been staff nurse at No. 4 Redlands War Hospital, Reading, and sister-in-charge at the Auxiliary Military Home Hospital, Radcliffe, Lancs. Miss Roskell has also done private nursing.

Willesden Infirmary.—Miss Annie Gertrude Farrington has been appointed superintendent night nurse. She was trained at Lake Hospital, Ashton-under-Lyne, and at Queen Charlotte's Hospital, has been staff nurse at the Garrett Anderson Hospital, and sister at the Royal Cornwall Infirmary. Miss Farrington holds the certificate of the Central Midwives Board.

Borough of Harrogate.—Miss Margaret J. Wrather has been appointed school nurse and health visitor. She was trained at the Royal Infirmary, Bradford, has been in charge of infant-welfare work at the Hostel, Derby, and health visitor for the County Borough of Derby. Miss Wrather holds the certificates of the Central Midwives Board and the Royal Institute of Public Health.

Borough Isolation Hospital, East Ham.—Miss Christine N. J. Davidson has been appointed sister. She was trained at Glasgow Royal Infirmary, from thence went to Ruchill Fever Hospital, Glasgow, and later was sister at Townley's Auxiliary Military Hospital, Bolton, and at Monsall Fever Hospital, Manchester.

Edmonton Union Infirmary, Tottenham.—Miss Dorothy Mary Parker, Miss Mary Martin, and Miss Isabella Smith have been appointed sister, night sister, and maternity sister respectively. Miss Parker was trained at the Royal Hospital, Portsmouth, and has done military nursing in England and overseas. Miss Martin was trained at Mile End Infirmary, has done private nursing, and has served in the Territorial Force Nursing Service at Manchester. Miss Smith was trained at St. Marylebone Infirmary, and has been sister-tutor at the East End Lying-in Hospital.

Farnham Union Infirmary.—Mrs. Ella A. Hudson has been appointed sister. She was trained at the Ashton-under-Lyne Infirmary, and in midwifery at the Jessop Hospital, Sheffield, and has been charge nurse at Wellingborough Infirmary.

Royal Alexandra Children's Hospital, Rhyl.—Miss Alice Edith Bright has been appointed lady superintendent. She was trained at the Queen's Hospital for Children, Hackney Road, London, has been theatre and ward sister in the Jenny Lind Hospital for Sick Children, Norwich, and has since been sister-in-charge of hospital wards and theatre at the Royal Alexandra Hospital, Rhyl.

Borough Hospital, Bedford.—Miss Fanny Ashton has been appointed sister. She was trained at Dewsbury General Infirmary, and has been staff nurse in the Territorial Force Nursing Service, sister at Northampton Tuberculosis Hospital, at Cambridge Nursing Hostel, and of the children's ward at Stockport Infirmary. She has also done private nursing.

County Hospital, York.—Miss E. Violet Arlioge has been appointed night sister. She was trained at St. Bartholomew's Hospital, where she later took temporary sister's duties. She has since been sister at the Royal National Hospital for Consumption, Ventnor, and sister-in-charge of Bradford Auxiliary Hospital.

Ty-Bryn Infirmary, Tredegar.—Miss Louisa Harriett Berry, A.R.R.C., has been appointed ward sister. She was trained at Farnham Infirmary, and has since done private nursing, and has been night sister at the Royal Victoria Hospital, Folkestone. She has also served in the Territorial Force Nursing Service for five years as sister, at home, and in Malta, France, and Italy.

Isolation Hospital, Winchmore Hill. — Miss Janet McFadyn has been appointed matron. She was trained at Leith Hospital, Edinburgh, and at Ruchill Hospital, Glasgow. She has since been night superintendent at Gateside Hospital, Greenock, at East Ham Borough Hospital, and assistant matron of the Cancer Hospital since 1914.

### Forthcoming Events.

Home Hospitals Association, Fitzroy Square, W.—The forty-second ordinary general meeting will be held at Fitzroy House, 16, 17, and 18 Fitzroy Square, London, W., on Friday, March 19, at 5 p.m., Sir John Bland Sutton, F.R.C.S., in the chair.

Poplar Hospital for Accidents.—Annual General Meeting of Governors at the Hospital, Tuesday, March 23, 1920, at 3.30 p.m.

### PRUDENTIAL ASSURANCE COMPANY Ltd.

CHIEF OFFICE—HOLBORN BARS, LONDON, E C. 1.

Summary of the Report presented at the Seventy-First Annual Meeting, held on March 4th, 1920.

ORDINARY BRANCH.—The number of policies issued during the year was 138,037, assuring the sum of £22,319,642, and producing a new annual premium income of £1,639,762. The premiums received were £7,627,547, being an increase of £856,708 over the year 1918.

The claims of the year amounted to £5,267,396, of which £175,882 was in respect of War Claims. The number of deaths was 12,829. The number of endowment assurances matured was 33,367, the annual premium income of which was £174,225.

The number of policies, including annuities, in force at the end of the year was 1,043,309.

INDUSTRIAL BRANCH.—The premiums received during the year were £11,155,874, being an increase of £1,419,471.

The claims of the year amounted to £3,997,138, of which £321,178 was in respect of 19,562 War Claims. The total number of claims and surrenders, including 36,260 endowment assurances matured, was 405,709.

The number of free policies granted during the year to those policyholders of five years' standing and upwards who desired to discontinue their payments was 72,293, the number in force being 2,036,395. The number of free policies which became claims was 50,209.

The total number of policies in force in this Branch at the end of the year was 23,097,157 : their average duration exceeds fourteen years.

The War Claims of the year, in both Branches, number 21,661, and amount to £497,060. The total paid up to the present on this account since the outbreak of War exceeds £5,300,000, in respect of 249,000 claims.

GENERAL BRANCH.—In March 1919 the Company commenced to transact Fire, Accident, and other classes of Insurance business. The premiums received during the year, after deducting reinsurances, amounted to £84,583. In addition, Sinking Fund policies have been issued insuring a capital sum of £305,825 and producing an annual income of £7,470.

Policies have been issued covering loss from Fire, Accident, Employers' Liability, Burglary, Plate Glass, Motor, Third Party, Lift, and other risks. The public are evincing special interest at the present time in the "Hearth and Home" Policy and the "Combined Fire and Burglary" Policy.

The assets of the Company, in all branches, as shown in the balance sheet, after writing down book values by £2,543,000, are £117,739,336, which, after deduction of the balance of £3,500,000 owing in respect of the advance from our Bankers for purchase of War Loan, shows an increase of £5,112,474 over 1918.

In the Ordinary Branch the surplus shown is £1,803,709, including the sum of £149,670 brought forward from last year and £500,000 transferred from the Contingency Fund. Out of this surplus the Directors have added £593,000 to the Investments Reserve Fund, which, after writing down book values by £1,443,000, stands as at 31st December, 1919, at £1,800,000, and £152,003 has been carried forward.

The Directors are pleased to be able to announce that a bonus of £1 8s. per cent. on the original sums assured will be allocated to participating policies in the Ordinary Branch which were in force on the 31st December, 1919

During the War, owing to the pressure on the Staff and the need for conserving paper supplies, no bonus certificates could be distributed. This year a certificate will be sent to every participating policyholder showing the Bonus now declared and the total bonuses added to their policies, and calling attention to the fact that bonuses of £1 per cent. were distributed in respect of each of the years 1915, 1916, and 1917, and a bonus of £1 6s. per cent. in respect of 1918.

In the Industrial Branch the surplus shown is £867,489, including the sum of £70,885 brought forward from last year. Out of this surplus the Directors have added £400,000 to the Investments Reserve Fund, which, after writing down book values by £1,100,000, stands as at 31st December, 1919, at £1,100,000, £100,000 has been carried to the Common Contingency Fund, and £72,394 has been carried forward.

The provisions of the Courts (Emergency Powers) Act are still in force, and the reserve of £100,000 is retained to meet losses from this cause to which the Company is still exposed.

The total surplus of the two branches, as shown by the valuation, is £2,671,198. Of this amount £593,000 has been added to the Investments Reserve Fund of the Ordinary Branch, £400,000 has been added to the Investments Reserve Fund of the Industrial Branch, and £100,000 to the Common Contingency Fund; £953,801 will be allocated to participating policies of the Ordinary Branch and £400,000 to the holders of fully paid shares in accordance with the Articles of Association of the Company, leaving £224,397 to be carried forward, namely, £152,003 in the Ordinary Branch and £72,394 in the Industrial Branch.

In the General Branch, the new classes of business have not been in operation for a full year, the existing contracts having on the average about eight months to run. The profit-earning capacity of the business therefore has not yet been ascertainable, and it has been considered advisable to retain 55 per cent. of the premiums paid for the unexpired risks, and the whole of the balance of the fund as an additional reserve.

The aftermath of the Great War still affects the Company adversely, and during the year a sum of £497,060 was paid in War Claims. In addition, there has been further heavy depreciation in the values of securities, and the Company has had to carry no less than £993,000 to the Investments Reserve Fund, and £100,000 to the Common Contingency Fund. There is thus a total of £5,543,000 available to meet depreciation or for any other purpose. Of this amount £2,543,000 has been applied to writing down the values of securities, £100,000 has been added to the Common Contingency Fund, and the balance of £2,900,000 remains as Investments Reserve Funds. The Securities which have been written down are those which seem to offer little prospect of recovery to prices equivalent to our book values.

The four Prudential Approved Societies have during the year paid to their members benefits amounting to approximately £1,687,000, making a total of over £10,224,000 paid since National Insurance was introduced. The number of persons admitted to membership of the Societies during the year was 298,442, of whom 127,344 were men and 171,098 women.

During the year no fewer than 6,007 members of the Staff have resumed duty on demobilisation after Active Naval and Military Service. The Directors once again express their high appreciation of the National Service rendered by the Staff while with the Colours, and cordially welcome them on their return.

To those members of the Staff, men and women, who have carried on the business of the Company under very difficult conditions the Directors again tender their hearty thanks.

Messrs. Deloitte, Plender, Griffiths & Co. have examined the securities, and their certificate is appended to the balance sheets.

THOMAS C. DEWEY, *Chairman.*
J. IRVINE BOSWELL, } *Directors.*
W. J. LANCASTER, }
J. BURN, *Actuary.* A. C. THOMPSON,
G. E. MAY, *Secretary.* *General Manager.*

*The full Report and Balance Sheet can be obtained upon application.*

